# Post‐hypoxic status epilepticus – A distinct subtype of status epilepticus with poor prognosis

**DOI:** 10.1002/epd2.20164

**Published:** 2023-10-10

**Authors:** Kateriine Orav, Pilar Bosque Varela, Tanja Prüwasser, Lukas Machegger, Markus Leitinger, Eugen Trinka, Giorgi Kuchukhidze

**Affiliations:** ^1^ Department of Neurology, Member of the European Reference Network EpiCARE, Centre for Cognitive Neuroscience, Christian Doppler University Hospital Paracelsus Medical University of Salzburg Salzburg Austria; ^2^ Department of Neurology North Estonia Medical Centre Tallinn Estonia; ^3^ Department of Mathematics Paris‐Lodron University Salzburg Austria; ^4^ Department of Neuroradiology, Christian Doppler University Hospital Paracelsus Medical University of Salzburg Salzburg Austria; ^5^ Neuroscience Institute Christian Doppler University Hospital Salzburg Austria; ^6^ Karl Landsteiner Institute for Neurorehabilitation and Space Neurology Salzburg Austria

**Keywords:** EEG, global cerebral hypoxia, outcome

## Abstract

**Objective:**

To evaluate the clinical outcome of patients with possible and definitive post‐hypoxic status epilepticus (SE) and to describe the SE types in patients with definitive post‐hypoxic SE.

**Methods:**

Patients with definitive or possible SE resulting from hypoxic brain injury after cardiac arrest (CA) were prospectively recruited. Intermittent EEG was used for the diagnosis of SE according to clinical practice. Two raters blinded to outcome analyzed EEGs retrospectively for possible and definitive SE patterns and background features (frequency, continuity, reactivity, and voltage). Definitive SE was classified according to semiology (ILAE). Mortality and Cerebral Performance Categories (CPC) score were evaluated 1 month after CA.

**Results:**

We included 64 patients of whom 92% died. Among the survivors, only one patient had a good neurological outcome (CPC 1). No patient survived with a burst suppression pattern, low voltage, or electro‐cerebral silence in any EEG. Possible or definitive SE was diagnosed in a median of 47 h (IQR 39–72 h) after CA. EEG criteria for definitive electrographic SE were fulfilled in 39% of patients; in 38% – for electroclinical SE and in 23% – for ictal‐interictal continuum (IIC). The outcome did not differ significantly between the three groups. The only patient with good functional outcome belonged to the IIC group. Comatose non‐convulsive SE (NCSE) without subtle motor phenomenon occurred in 20% of patients with definitive electrographic SE and outcome was similar to other types of SE.

**Significance:**

Possible or definitive SE due to hypoxic brain injury is associated with poor prognosis. The outcome of patients with electrographic SE, electroclinical SE, and IIC did not differ significantly. Outcome was similar in patients with definitive electrographic SE with and without prominent motor features.

**Key points**

- Possible and definitive status epilepticus (SE) due to hypoxic brain injury is associated with high mortality and poor functional outcome.
- The outcome of patients with post‐hypoxic electrographic SE, electroclinical SE, and ictal‐interictal continuum did not differ significantly.
- A purely non‐motor phenotype of non‐convulsive SE occurred in 20% of patients, and outcome was similar to other types of post‐hypoxic SE.

## 1 INTRODUCTION

Status epilepticus (SE) is a life‐threatening condition with a wide spectrum of etiologies.^1^ SE occurs in up to a third of comatose patients after cardiopulmonary resuscitation (CPR) and is associated with worse outcome compared to SE caused by other etiologies.^2^, ^3^, ^4^ Yet with advances in resuscitation and intensive care, several reports have emerged of the prognosis not being uniformly bad with at least 10% of patients achieving meaningful neurological recovery after SE.^5^, ^6^ Coma with non‐evolving periodic EEG patterns has been listed as a currently indeterminate or boundary condition in the ILAE classification of SE.^1^ However, it cannot be excluded that some rhythmic and periodic patterns at lower frequencies in post‐hypoxic patients could also reflect a potentially ictal state and not only be markers of underlying brain injury.^7^, ^8^ Whether outcome differs between patients with rhythmic and periodic patterns with a frequency of more and <2.5 Hz is not clear.

Effort has been made to identify characteristics of post‐hypoxic SE that may influence prognosis. Several EEG background characteristics such as burst suppression, suppressed background activity, and absent reactivity in isolation or combination with SE have shown to carry a worse prognosis.^9^, ^10^, ^11^ However, SE and associated EEG patterns alone are insufficient to prognosticate outcome in patients after cardiac arrest (CA) and should be used as part of multimodal prognostication.^12^, ^13^

Different SE types have been associated with varying prognostic implications in non‐hypoxic SE.^14^ Outcome may be better in patients without motor phenomenon with favorable outcome reported in ¼ of patients with non‐convulsive SE (NCSE) after cardiac arrest.^15^ Myoclonus as motor phenomena occurs often in patients with post‐hypoxic SE and is associated with worse outcome but not invariably so.^16^, ^17^ The timing of onset of SE after hypoxic brain injury may also play an important role with earlier onset after CA having been associated with worse outcome, possibly as a sign of more extensive underlying brain damage.^6^

The aim of our study was to evaluate the clinical outcome of possible and definitive post‐hypoxic SE and to describe the SE types in patients with definitive post‐hypoxic SE.

## 2 METHODS

### 2.1 Study cohort

Patients with definitive or possible SE were prospectively recruited between 20.02.2019 and 17.03.2023 through the EEG laboratory of Christian Doppler University Hospital, Paracelsus Medical University of Salzburg, Austria. For this study, all patients with definitive or possible SE resulting from hypoxic brain injury after CA were included from the SE database.

### 2.2 Standard post‐cardiac arrest care and multimodal prognostication

According to local guidelines, all resuscitated patients who did not regain consciousness after return of spontaneous circulation (ROSC) underwent targeted temperature management (TTM) to a goal of 32–36°C for 24 h followed by controlled rewarming. During TTM, patients received analgo‐sedation with propofol and remifentanil. As part of multimodal neuroprognostication, (1) absent pupillary reflexes, (2) elevated neuron‐specific enolase (NSE) levels, (3) bilaterally absent median nerve somatosensory evoked potentials (SSEPs), (4) “highly malignant” EEG patterns (such as burst suppression, low voltage EEG, periodic discharges with suppressed background), and (5) severe ischemic injury on brain imaging were considered negative prognostic markers. If ≥3/5 occurred, a negative prognosis was deemed likely and withdrawal of life‐sustaining therapy (WLST) could be considered earliest at 72 h after cardiac arrest.^18^ In patients with SE, antiseizure therapy was implemented according to treating physician's discretion if other prognostic markers did not unanimously indicate a negative prognosis.

### 2.3 Clinical variables

A retrospective chart review was performed to collect the following variables: age, sex, baseline modified Rankin score (mRS), acute comorbidities (acute myocardial infarction, acute renal failure, pneumonia or others), CA type (primary cardiac or non‐cardiac/unknown), primary cardiac rhythm (shockable or non‐shockable), place of CA (in‐ or out‐of‐hospital), time to ROSC, bystander CPR initiation, TTM therapy, sedation and analgesia details, use of antiseizure medications (ASM), pupillary and corneal reflexes (absent/present), SSEPs (present/absent bilaterally), peak serum NSE level within 72 h, CT/MRI findings (normal/abnormal), and days in ICU. Occurrence of clinical seizures or motor phenomena was identified from clinical notes and EEG reports.

### 2.4 EEG acquisition and classification

Intermittent EEG was performed as part of standard care in patients who remained comatose after stopping sedation or if there was a clinical suspicion of seizures. EEG was acquired using 21 electrodes placed according to the 10–20 international system with a minimal duration of 20 min.^19^ According to local protocol, EEG should be performed within 72 h in post‐hypoxic comatose patients. Repeated EEGs were performed if clinically indicated. Reactivity was tested through auditory, tactile, or noxious stimulation. EEG recordings were interpreted by board‐certified experts during standard care and communicated to the intensive care physicians. Treatment decisions regarding ASM and sedation were made at treating physicians' discretion after discussion with the EEG specialist.

Two independent raters (P.B.V. and G.K.), blinded to outcome, retrospectively evaluated the EEGs for background activity, rhythmic or periodic patterns (periodic discharges [PD], spike and wave [SW] patterns or lateralized rhythmic delta activity [LRDA]), plus modifiers, fluctuation, and reaction to treatment trial (if performed) according to the American Clinical Neurophysiology Society (ACNS) standardized critical care EEG terminology.^20^ Background activity was evaluated for frequency, continuity, reactivity, and voltage. Patterns with <50% of the recording consisting of suppression were considered discontinuous and patterns with ≥50% suppression as burst suppression. In case of burst suppression, the bursts were classified as identical or non‐identical. Reactivity was evaluated as any change in background frequency or amplitude after stimulation. EEG was graded as low voltage if activity throughout the recording was <20 μV and electro‐cerebral inactivity was stated if no EEG activity over 2 μV was discernible. EEG reports were used to evaluate whether myoclonus was time‐locked to EEG bursts or epileptiform discharges as videos were not available.

The EEGs were classified as definitive electrographic SE, definitive electroclinical SE, possible electrographic SE/ictal‐interictal continuum (IIC), or no SE according to the ACNS and Salzburg criteria.^20^, ^21^, ^22^ In cases where disagreement existed between the two raters about whether EEG fulfilled criteria of possible or definitive electrographic SE, consensus was achieved with a third reviewer (M.L.). Definitive SE was classified according to semiology based on the 2015 ILAE classification.^11^ Subtle myoclonus isolated to one body part was considered as a subtle motor feature, and SE was accordingly classified as non‐convulsive SE (NCSE) in coma.

### 2.5 Outcomes

Survival and Cerebral Performance Categories (CPC) score (CPC 1 – no neurological disability; CPC 2 – moderate disability; CPC 3 – severe disability, CPC 4 coma or minimal conscious state; CPC 5 – death) were assessed at 30 days.

### 2.6 Statistical analysis

The data were analyzed using R version 4.1.3. The patients' demographic and clinical characteristics were summarized using descriptive methods. For estimating the odds ratio, the conditional maximum likelihood estimator was used. Fisher's exact test was used for group comparisons. Interrater agreement for EEGs fulfilling SE criteria was tested using Cohen's kappa coefficient.

## 3 RESULTS

Sixty‐five patients with possible or definitive post‐hypoxic SE were identified among the 557 patients recruited in the SE database (11.5%). The interrater agreement for EEG interpretation was very high (*κ* = .92) with 97.5% agreement for diagnosing possible or definite electrographic SE. After resolving disagreement regarding three EEGs, one patient was excluded due to not fulfilling criteria for definitive or possible electrographic SE.

### 3.1 Clinical characteristics and outcome

Within 30 days after CA, 59/64 (92%) patients with post‐hypoxic possible or definitive SE died. Among five survivors, only one patient achieved good functional neurological outcome (CPC 1). The functional outcome of the other survivors was CPC 3 in three patients and CPC 4 in one patient. Patients' clinical characteristics did not differ significantly between surviving and non‐surviving patients (Table 1). In our cohort, a primary shockable rhythm was detected in 28/64 (44%) patients and a primary cardiac cause was the reason for CA in 36/64 (56%) patients. The median time to ROSC was not significantly longer in surviving patients than non‐surviving patients (42.5 vs. 20 min). All surviving patients had preserved brainstem reflexes and SSEPs.

**TABLE 1:** Clinical characteristics of the study population grouped by outcome.

	Survivors (*n* = 5), *n* (%)	Non‐survivors (*n* = 59), *n* (%)	Total (*n* = 64), *n* (%)
Age (year), median [IQR]	63 [57–64]	67 [62–75]	66 [62–74]
Females	3 (60)	19 (32)	22 (34)
Baseline mRS, median [IQR]	0 [0–0]	0 [0–0]	0 [0–0]
Acute comorbidities			
0	0 (0)	19 (32)	19 (30)
1	1 (20)	22 (37)	23 (36)
2	2 (40)	12 (20)	14 (22)
≥3	2 (40)	6 (10)	8 (13)
Shockable rhythm	4 (80)	24 (41)	28 (44)
Cardiac cause of CA	4 (80)	32 (54)	36 (56)
Out of hospital CA	5 (100)	47 (80)	52 (81)
Bystander CPR	3/5 (60)	37/54 (69)	40/59 (68)
Time to ROSC (min), median [IQR]	42.5 [34–49]	20 [15–30]	20 [15–32]
TTM	5 (100)	52/58 (90)	57/63 (90)
NSE (μg/L), median [IQR]	80 [58–84]	70 [41–144]	70.5 [42–124]
Absent brainstem reflexes	0/5 (0)	9/44 (20)	9/48 (19)
Bilaterally absent SSEPs	0/5 (0)	17/49 (35)	17/54 (31)
Abnormal imaging (MRI/CT)	2/5 (40)	28/56 (50)	30/61 (49)
No. of ASM, median [IQR]	2 [1–2.5]	2 [1.5–3]	2 [1–3]
Benzodiazepines	4 (80)	49 (83)	54 (84)
No. of sedatives, median [IQR]	3 [1–3]	1 [1–1]	1 [1–1]

*Note*: No statistical significance in variables was determined between the two groups.

Abbreviations: CA, cardiac arrest; CPR, cardiopulmonary resuscitation; mRS, modified Rankin score; ROSC, return of spontaneous circulation; SSEPs, somatosensory evoked potentials; TTM, targeted temperature management.

The median number of ASM used per patient was two (IQR 1–3). The following ASM were used in our cohort: levetiracetam (98%), valproate (58%), lacosamide (45%), and others (perampanel and phenytoin; each in 14%). In 85% of patients, benzodiazepine boluses were administered during treatment. The following benzodiazepines were used: lorazepam (64%), clonazepam (19%), midazolam (8%), diazepam (2%). The most commonly used sedatives were propofol (98%), followed by midazolam and ketamine (both used in 13% of patients). ASM and sedative use did not differ significantly between the groups.

### 3.2 EEG baseline characteristics and outcome

A median of 2.5 (IQR 2–4) EEGs was registered per patient with the first EEG recorded in a median of 46 h (IQR 35.5–63.5 h) after CA. EEG baseline characteristics in survivors and non‐survivors are shown in Table 2. No patient with a burst suppression pattern (regardless if bursts were identical or non‐identical), low voltage, or electro‐cerebral silence in any EEG, survived. Background activity, even though always abnormal (at least moderately slowed and/or low voltage), was seen in all surviving patients but in only 24/59 (41%) of non‐survivors. Reactivity to at least one exogenous stimulus was seen in 2/5 (40%) patients who survived compared to 7/59 (12%) patients who did not. The EEG baseline characteristics did not differ statistically between survivors and non‐survivors.

**TABLE 2:** EEG background characteristics of definitive or possible electrographic status epilepticus grouped by outcome.

	Survivors (*n* = 5), *n* (%)	Non‐survivors (*n* = 59), *n* (%)	Total (*n* = 64), *n* (%)
Background present	5 (100)	24 (41)	29 (45)
Discontinuous pattern	2 (40)	10 (17)	12 (19)
Burst suppression			
Identical bursts	0 (0)	9 (15)	9 (14)
Non‐identical bursts	0 (0)	8 (14)	8 (13)
Electro‐cerebral silence/low voltage	0 (0)	11 (19)	11 (17)
Reactivity present	2 (40)	7 (12)	9 (14)

*Note*: No statistical significance in variables was determined between the two groups.

### 3.3 SE and IIC EEG patterns and outcome

Possible electrographic or definitive SE was identified in a median of 47 h (IQR 39–72 h) after CA. All five survivors were diagnosed with possible or definitive SE >48 h after CA. In 7/64 (11%) patients, the first EEG did not show a possible or definitive SE pattern, but it was diagnosed on a subsequent recording. In these patients, possible or definitive SE was preceded by a burst suppression pattern in three patients, low voltage EEG in two patients, discontinuous pattern in one patient, and continuous generalized theta activity in one patient. Twenty‐five out of 64 (39%) patients fulfilled EEG criteria for definitive electrographic SE, 24/64 (38%) were classified as electroclinical SE (i.e. motor phenomenon occurred) and 15/64 (23%) fulfilled criteria for IIC (possible electrographic SE). EEG characteristics of the groups are presented in Table 3. One (4%) patient survived in both the electrographic and electroclinical SE group with both patients achieving a CPC 3 score at 1 month after CA. The EEG patterns in these two patients were GPD >2.5 Hz and GPD 1.0–2.5 Hz respectively. Three (20%) patients in the IIC group survived. The only patient with good neurological outcome (CPC 1) had an EEG pattern classified as IIC (GPDs .5–1 Hz + R). In this patient, the IIC pattern developed from a continuous background and was identified 89 h after CA, while no IIC pattern occurred 44 h after CA. The other two patients with IIC who survived had EEG patterns of GPD 1.0–2.5 Hz and LRDA >1 Hz with plus modifier and CPC 3 and CPC 4 scores respectively at 1 month after CA.

**TABLE 3:** EEG patterns of patients with post‐hypoxic electrographic SE, electroclinical SE, and IIC.

ED >2.5 Hz	*n* (%)	ED <2.5 + clinical phenomenon	*n* (%)	IIC	*n* (%)
*n* = 25		*n* = 24		*n* = 15	
GPD	20 (80)	GPD	19 (79)	GPD 1.0–2.5 Hz	11 (73)
				BIPD 1.0–2.5 Hz	1 (7)
S/SW	5 (20)	Burst suppression	4 (17)	GPD .5–1 Hz with plus modifier/fluctuation	1 (7)
		BIPD	1 (4)	LRDA >1 Hz with plus modifier/fluctuation	2 (13)

Abbreviations: BIPD, bilateral independent periodic discharges; ED, epileptiform discharges; GPD, generalized periodic discharges; IIC, ictal‐interictal continuum; LRDA, lateralized rhythmic delta activity; S/SW, spike and sharp wave.

Treatment with ASM resulted in an improvement of the EEG pattern in 18/21 (86%) patients with electrographic SE, in 18/20 (90%) patients with electroclinical SE, and in 9/11 (82%) patients with IIC. In patients with electrographic SE, the SE persisted on follow‐up EEGs in 8/25 (32%) patients. SE evolved into an IIC pattern in 9/25 (36%) patients. In 2/25 (8%) patients, SE evolved first into an IIC pattern and then into a no SE pattern. Electrographic SE had resolved in 3/25 (12%) patients with a low‐voltage EEG seen on a follow‐up. In 3/25 (12%) patients with electrographic SE, a follow‐up EEG was not performed.

### 3.4 Motor phenomenon and SE classification

Motor phenomena occurred in 44/64 (69%) patients in the whole cohort. Myoclonus was focal in 68% (30/44) of patients and generalized in 32% (14/44) of patients. In patients fulfilling criteria for definitive electrographic SE, motor phenomena occurred in 20/25 (80%) patients. As per definition, all patients categorized as electroclinical SE had motor phenomena. Among patients with focal or generalized myoclonus, in 34/44 (78%) cases, the myoclonic jerks were time‐locked with epileptiform discharges on EEG. In the remaining 10 patients, myoclonus did not occur during EEG or the association with epileptiform discharges was not evaluated.

Among patients with definitive electrographic SE, SE was classified as myoclonic SE (with prominent epileptic myoclonic jerks) in 3/25 (12%) patients, convulsive (tonic–clonic) to NCSE in 4/25 (16%) patients, and NCSE in coma in 18/25 (72%) patients. In the NCSE group, 13/18 (72%) patients had subtle motor signs, mainly consisting of isolated ocular or facial myoclonus. All four patients with convulsive NCSE also had subtle myoclonia during the treatment period. In patients classified as electroclinical SE, 10/24 (42%) had generalized myoclonus and 14/24 (58%) had focal myoclonus.

### 3.5 Withdrawal of life‐sustaining therapies

Death occurred as a result of WLST primarily due to overt pessimistic neurological prognosis in 51/59 (86%) patients and due to medical reasons (acute comorbidities or progressive chronic comorbidity) in 6/59 (10%) patients. Brain death was declared in 2/59 (3%) patients. The median time of death in patients with WLST due to poor neurologic prognosis was 8 days (IQR 7–11 days) after CA.

Among non‐survivors, 9/59 (15%) patients had otherwise favorable prognostic profiles according to current guidelines (NSE <60 μg/L, brainstem reflexes and SSEPs present as well as non‐remarkable brain imaging).^13^ EEG background characteristics in these patients were as follows: no patients had low voltage EEG or a burst suppression pattern, 2/9 patients had discontinuous EEG background activity, in 3/9 patients background reactivity was present. Six of these patients fulfilled EEG criteria for definitive SE, two patients belonged to the electroclinical SE group and one patient had an IIC pattern. In the six patients with electrographic SE, SE was classified as NCSE in 5/6 (two had subtle motor phenomenon) and convulsive to NCSE in 1/6 patients. SE EEG pattern was spikes/sharp waves >2.5 Hz in 3/6 patients and GPD >2.5 Hz in 3/6 patients. In the three patients with electroclinical SE or IIC, the EEG pattern was GPD 1–2.5 Hz. All nine patients received sedation with propofol, benzodiazepines, and a median of two ASMs (IQR 2–2.5). In patients with electrographic SE, the SE persisted in 1/6 patients, SE resolved in 1/6 patients, SE evolved into an IIC pattern, and then low voltage EEG in 1/6 patients and SE evolved into an IIC pattern in 3/6 patients. Two out of nine (22%) patients with otherwise favorable prognostic profiles died of acute comorbidities (one with electroclinical SE and one with IIC), and the remaining 7/9 (78%) died as a result of WLST a median of 11 days (IQR 10–14.5) after CA. The decision of WLST in these patients was influenced by a combination of presence of pre‐existing comorbidities and if known, patient's wishes.

## 4 DISCUSSION

In this study, we found a poor outcome with a 30‐day mortality of 92% in patients with possible or definitive post‐hypoxic SE. More so, only one of the five surviving patients achieved good functional neurological outcome. Our results are in line with previous studies that have shown SE to be a strong and independent predictor of mortality and poor functional outcome after CA.^3^, ^23^ However, some of these studies reported a more favorable outcome with 10%–44% of patients making a meaningful neurological recovery.^5^, ^6^, ^24^ There are several possible reasons for worse outcome in our patients. Firstly, compared to previous cohorts, our patients more frequently had baseline characteristics, which are independently associated with worse outcome (non‐shockable initial rhythm and non‐cardiac cause of CA).^25^, ^26^ Surviving patients also had a markedly prolonged time to ROSC, which may have influenced the poor neurological outcome. Secondly, not all post‐hypoxic SE were alike. Over 50% of our patients had at one point EEG patterns independently associated with worse outcome such as burst suppression or discontinuous pattern, low voltage, or electro‐cerebral silence.^9^, ^27^, ^28^, ^29^, ^30^ The occurrence of these malignant EEG patterns increases the negative predictive value of post‐hypoxic SE.^10^, ^31^ Time of onset of SE may also be an important factor, with a SE onset within 36 h after CA associated with poor outcome, possibly as a marker of more extensive brain injury.^6^ Due to the use of intermittent EEG in our study, the exact time of possible or definitive SE onset could not be evaluated. However, SE diagnosed in a median of 47 h after CA in our cohort indicates that at least half of the patients had a SE, which developed in the earlier phase after CA. While the predictive value of EEG has been shown not to improve more than 24 h after CA in patients who had early EEG, our results confirm a pessimistic prognosis in patients with malignant EEG patterns at later time points.^9^, ^32^, ^33^ We were not able to analyze the doses of sedative medications due to incomplete data. While sedatives are known to affect EEG, they have not been shown to influence the prognostic value of unfavorable EEG patterns in patients with hypoxic brain injury.^10^, ^34^

There is uncertainty about which rhythmic and periodic patterns in post‐hypoxic patients reflect a “true” and reversible epileptic phenomenon. Patterns with a frequency <2.5 Hz may constitute a sign of severe and irreversible brain network injury as well as be related to metabolic disturbances which are commonly present in post‐hypoxic patients.^35^, ^36^ There is, however, evidence that GPDs can result from pathological activation of thalamo‐cortical networks similar to the generation of epileptiform discharges. Therefore, lower frequencies of GPDs may constitute a continuum between encephalopathy and status epilepticus.^37^ Periodic discharges have also been described to gradually increase in frequency in hours and days after CA making the diagnosis of post‐hypoxic SE more difficult than non‐hypoxic SE, especially with intermittent EEG.^6^, ^38^ Due to these reasons we have included patients with definitive and possible SE in our study. In our cohort of patients who remained comatose after cardiac arrest, 39% of patients fulfilled criteria for definitive electrographic SE, 38% for electroclinical SE, and 23% for IIC (possible electrographic SE). Outcome in all three groups was equally poor. The only patient with good functional neurologic outcome had an EEG pattern of GPDs .5–1 Hz with superimposed rhythmic activity consistent with the definition of IIC.

While aggressive treatment as early as possible is imperative in non‐hypoxic SE,^4^, ^39^ it remains uncertain whether the treatment of SE and EEG patterns not fulfilling criteria for definitive SE in post‐hypoxic patients influence outcome. Unstandardized and moderate‐intensity treatment, which is often used in clinical practice, has not been shown to improve outcome.^40^ Benzodiazepines were administered to 80% of our patients in addition to a median of two ASM. There was no significant difference between ASM and benzodiazepine use in patients with electrographic SE, electroclinical SE, and IIC. The recently published TELSTAR trial evaluating standardized treatment of rhythmic and periodic EEG activity of any frequency did not show improved outcome compared to standard care. However, exploratory subgroup analysis suggested that patients with non‐GPD patterns may benefit from aggressive treatment.^38^ In our cohort, 9/64 (14%) patients had non‐GPD patterns. Only one of these patients with LRDA survived but had a poor neurological outcome (CPC 3).

In our cohort, definitive electrographic SE was classified as non‐convulsive in 72%, convulsive to NCSE in 16% and with prominent motor symptoms (myoclonic SE) in 12% of patients. Our results differ from previous studies, which reported NCSE to occur in 15%–30% of patients with post‐hypoxic SE during sedation and TTM.^2^, ^6^, ^15^ The main reason for this variance is the different classification of patients with subtle motor features such as isolated eyelid myoclonia. While we have classified these patients as being in NCSE in coma, other authors have classified these patients as SE with prominent motor symptoms. In our cohort, over half of the patients classified as NCSE in coma had subtle motor features. A purely non‐motor phenotype of NCSE occurred in 20% of our patients, which is more in line with previous reports. It must also be considered that sedatives may mask clinical features of myoclonus and therefore generalized myoclonus may be transformed into a subtle or non‐motor phenotype.^41^ This further complicates SE classification based on motor phenomena in post‐hypoxic patients and may support the classification of patients with any myoclonus as SE with prominent motor features. Better outcome in patients with NCSE after hypoxic brain injury has been reported in a recent review indicating recovery in 24.5% of patients.^15^ Due to the low number of survivors in our cohort, we could not make associations between the type of SE and prognosis. The only patient who survived among patients with definitive electrographic SE was classified as convulsive to NCSE and had subtle myoclonus.

In the whole cohort, myoclonus occurred in the majority (69%) of patients. As myoclonus has been shown to be a predictor of poor prognosis in post‐hypoxic patients, this may be a further factor contributing to the poor outcome in our patients.^17^, ^42^ However, different clinical features of myoclonus may carry different prognosis. For example, multifocal myoclonus has been shown to be associated with better outcome than other types of myoclonus.^16^ Disagreement exists over whether myoclonus in post‐hypoxic patients is of cortical or subcortical origin.^43^ The first would support an epileptic etiology and the second more likely be a result of severe brain injury. To further complicate this issue, there is also no consensus regarding the diagnosis and classification of post‐hypoxic myoclonus. An important limitation of our study is the absence of video recordings for electro‐clinical correlation of myoclonus and EEG patterns. We have used EEG reports and clinical descriptions to evaluate the association of EEG and motor features as well as clinical myoclonus characteristics. This may have led to bias toward the electro‐clinical association of post‐hypoxic myoclonus.

Due to the dismal outcome of our cohort, we looked in more detail at a group of nine patients who otherwise had favorable prognostic markers and therefore did not fulfill ≥2 negative prognostic criteria indicative of a likely poor prognosis. These patients had favorable EEG background characteristics. Two‐thirds of these patients fulfilled EEG criteria for definitive SE. They received sedation with propofol, benzodiazepines, and a median of two ASMs. Yet none of these patients awakened and death due to WLST occurred in a median of 11 days after CA. As clinicians were not blinded to EEGs, we cannot exclude that the EEG findings could have influenced a presumed futile neurologic prognosis and contributed to a self‐fulfilling prophecy. Late awakening after post‐hypoxic coma has been described in one‐third of patients in a median of 5 days after stopping sedation, however with a range of 3–23 days.^44^ Therefore, it cannot be ruled out that single outliers could have benefitted in terms of survival from prolonging therapy in our cohort. It is not known how long the optimal time to continue life‐sustaining therapy in such patients is. The European Resuscitation Council and the European Society of Intensive Care Medicine guideline points out that while late awakening does not preclude neurological recovery the likelihood of awakening and making neurological recovery decreases progressively in time after resuscitation.^12^, ^45^

Our study has several other limitations including retrospective data analysis. The rarity of good outcome within the cohort did not allow proving statistically significant differences between the groups. The treating clinicians were not blinded to any of the prognostic test results including EEG. Finally, the use of intermittent EEG limited the evaluation of the onset and duration of possible or definitive SE but portrays the standard clinical practice of EEG use in post‐hypoxic patients in many centers.

## 5 CONCLUSION

Possible or definitive status epilepticus after CA is associated with poor prognosis in patients with hypoxic brain injury. The outcome of patients with electrographic SE, electroclinical SE, and IIC did not differ significantly. The classification of post‐hypoxic SE is complex with uncertainty regarding myoclonus especially if subtle. A purely non‐motor phenotype of NCSE occurred in 20% of patients, and outcome was similar to other types of SE. Prospective studies are warranted to identify whether and which definitive and possible post‐hypoxic SE patients could benefit of prolonged and more aggressive therapy.

6

**Test yourself**

1. Which of the following EEG patterns is associated with a better outcome in a status epilepticus following global cerebral hypoxia after cardiac arrest?
  - Burst suppression
  - Electro‐cerebral silence
  - Preserved reactivity
  - Low‐voltage EEG
  - Absence of background activity
2. A trend towards lower mortality is associated with:
  - Non‐convulsive status epilepticus without motor features
  - Non‐convulsive status epilepticus with subtle motor signs
  - Myoclonic status epilepticus
  - Convulsive to non‐convulsive status epilepticus
  - Convulsive status epilepticus
3. Which of the following neuroprognostication markers are NOT utilized in patients with a status epilepticus due to hypoxic brain injury after cardiac arrest:
  - Absent pupillary reflexes
  - Elevated neuron‐specific enolase (NSE) levels
  - Bilaterally absent visual evoked potentials (VEP)
  - Malignant EEG patterns
  - Severe ischemic injury on brain imaging

Answers may be found in the *Supporting information*

## Supporting information


Appendix S1.
