# Supplementary material for: Post‐hypoxic status epilepticus – A distinct subtype of status epilepticus with poor prognosis
Source: Epileptic Disord. 2023 Oct 10;25(6):823–32. doi: 10.1002/epd2.20164 (PMC10947449; doi:10.1002/epd2.20164)
Supplement: Supplementary file 1 — Appendix S1. [file EPD2-25-823-s001.docx]

Correct answers:
1. c
2. a
3. c
